# The relationship between social avoidance, behavioral self-regulation, and social adjustment among Chinese Kindergartners

**DOI:** 10.1186/s40359-025-03063-7

**Published:** 2025-09-01

**Authors:** Jingjing Zhu, Yu Shen, Yan Li

**Affiliations:** Shanghai Institute of Early Childhood Education, Shanghai Normal University, 100 Guilin St, 200234, Shanghai, China

**Keywords:** Social avoidance, Social adjustment, Behavioral self-regulation, China, Kindergartners

## Abstract

Research consistently indicates that socially avoidant children face challenges in adapting to social settings. This investigation aimed to explore the relationship between social avoidance and social adjustment and the moderating role of behavioral self-regulation. The study included 211 children (111 boys, *Mage* = 48.81 months, *SD* = 2.51). These children attended two public kindergartens located in Shanghai, China. The current study also include data from children’s teachers and mothers. After controlling for child gender, age, shyness, unsociability, and parental education level, the research found that: (1) Social avoidance was positively associated with asocial behavior when behavioral self-regulation was low, but negatively associated when behavioral self-regulation was high; (2) Peer exclusion was unrelated to social avoidance at low behavioral self-regulation, but negatively associated at high behavioral self-regulation; (3) Anxious-fearful behavior showed a similar pattern, with a significant negative association emerging only at high behavioral self-regulation. These results suggest that higher behavioral self-regulation may buffer or alter the interpretation of socially avoidant behavior.

## Introduction

Substantial empirical evidence from investigations into children's social adjustment demonstrates that early deficits in social competence may precipitate a constellation of deleterious outcomes across an individual's subsequent developmental trajectory [50]. Social adjustment constitutes a dynamic process whereby individuals progressively acquire and modify behavioral repertoires and lifestyle patterns through reciprocal interactions with their social environment, ultimately facilitating congruence and homeostasis within their sociocultural context [46]. Contemporary developmental researchers predominantly assess social adjustment in preschool-aged children through systematic examination of three fundamental domains: social competence as the primary positive indicator, externalizing problems (e.g., aggressive behavior, oppositional tendencies) and internalizing problems (e.g., anxious symptoms, social withdrawal) as negative indicators of adjustment (e.g., [49, 72]). Building upon this conceptual framework, the current empirical investigation focuses on three specific dimensions central to children's interpersonal functioning that map onto these broader domains: asocial behavior, peer exclusion, and anxious-fearful behavior. Children's asocial behavior, which indicates a preference for solitary play when in the company of peers [45], is a key manifestation of internalizing problems and also indicative of low social competence [66]. Peer exclusion, which encompasses experiences of being ignored, rejected, or ostracized by peers [45], serves as a critical negative outcome reflecting impaired social competence [44]. Anxious-fearful behavior, manifesting as heightened vigilance or wariness in peer interactions or unfamiliar social settings [45], falls squarely within the domain of internalizing problems [29].

Asendorpf [3] delineated three distinct types of social withdrawal: shyness, unsociability, and social avoidance. Rubin et al. [66] further noted that these constructs represent different manifestations of the broader phenomenon of social non-participation. Shyness encompasses both the desire for social communication and the apprehension about engaging in it. The primary characteristic of shy children manifests through heightened vigilance and internal anxiety when confronted with novel and unfamiliar situations [4]. On the other hand, unsociable children demonstrate diminished motivation toward sociability and latent propensities for social isolation. Such children exhibit minimal fear regarding social interactions. They neither initiate social engagement nor demonstrate interest in it, preferring instead to engage in activities that intrinsically appeal to them [21]. In contrast to being shy or unsociable, social avoidance characterized by a lack of motivation to engage with contemporaries and a tendency to actively avoid social interactions represents a more profound withdrawal from peers and adults. These children not only withdraw from peer interactions but also manifest fear in the presence of age-mates [3, 21]. This form of social withdrawal extends significantly beyond mere rejection in social relationships,it constitutes intentional avoidance of interactions [19]. The empirical outcomes associated with social avoidance appear to have more deleterious consequences than shyness and unsociability [21, 90]. For instance, compared to their peers, children exhibiting avoidance tendencies demonstrate the highest risk of psychosocial maladjustment and present concerning profiles in terms of peer relationships and aggression [21]. Research on preschool children indicates that social avoidance can be positively associated with peer exclusion, and internalizing problems, while negatively associated with interpersonal skills [25, 86]. Similarly, [20] found that social avoidance was uniquely associated with peer problems and depressive symptoms, even after controlling for shyness and unsociability. While numerous studies have investigated shyness and unsociability, research examining social avoidance among preschool children remains limited [24, 67]. This investigation aims to explore how social avoidance, as a discrete dimension of social withdrawal, influences critical aspects of social adjustment during early childhood.

Considering the developmental perspective with an emphasis on contextual factors, the cultural values internalized by children establish a framework for their evaluation and interpretation of specific behaviors. This cultural context significantly influences the development of these behaviors, altering both their manifestation and significance [13]. In individualistic Western societies, there is substantial emphasis on expressing personal attitudes and opinions, and passive or socially reticent behaviors are generally discouraged. This cultural orientation may result in more negative evaluations of socially withdrawn children [13]. Within traditional Chinese culture, children who demonstrate wariness, behavioral restraint, and self-regulation are often perceived as exhibiting social maturity, interpersonal competence, and insightfulness [75]. Consequently, shy Chinese children may experience greater social acceptance than their Western counterparts. For instance, [15] observed that characteristics of shyness were positively associated with leadership patterns in interaction and enhanced peer acceptance among Chinese children. In recent decades, Chinese society has undergone substantial transformations due to the comprehensive implementation of market economy reforms. Within the competitive urban environment, there has been increasing promotion of novel behavioral attributes such as cooperation and social engagement [51]. In this evolving social context, social avoidance may have distinct negative implications for children's adjustment and development. Research indicates that avoidant children received the most negative responses from their peers. They were anticipated to present the greatest challenges in the classroom, have the least favorable relationships with teachers, and be perceived as the least intellectually capable [13]. According to previous research, social withdrawal has been found to generate adjustment difficulties in Chinese adolescents [26, 27], and comparable findings have emerged in studies specifically examining early developmental periods [88, 90, 92, 91]. A study focused on Chinese preschool children revealed that not all children who exhibit asocial behavior or isolation experience difficulties with social integration [90]. Notably, investigations of social avoidance within the Chinese context are scarce [13] and have produced limited empirical evidence. Therefore, the primary objective of this article is to investigate the relationship between social avoidance and social adjustment within the Chinese cultural context.

Self-regulatory processes are fundamental to the development of personality and behavioral adjustment [10]. Children who exhibit social withdrawal demonstrate poorer behavioral self-regulation capabilities [89]. To the best of our knowledge, among the three manifestations of social withdrawal in childhood, social avoidance in Chinese children has received substantially less scholarly attention compared to their shyness and unsociability. Consequently, investigating the adaptive challenges that social avoidance may present constitutes an area that warrants further empirical investigation. This study aims to examine the relationship between social avoidance and social adjustment and to explore the moderating role of behavioral self-regulation in this association.

### Social avoidance and social adjustment

The detrimental effects of social avoidance persist throughout various developmental stages [7]. Substantial literature has demonstrated a significant association between social withdrawal and social adjustment in Western contexts. In examining research focused on preschool populations, [20] established that social withdrawal exhibited unique and substantial correlations with peer-related difficulties, which were associated with symptomatology corresponding to social phobia and depression. [60] determined that unsociable behavior toward peers mediates the relationship between solitary play preference and peer rejection,specifically, preference for solitary play positively predicts unsociable behavior toward peers, which subsequently positively predicts peer rejection. In investigations examining adolescent populations, [35] confirmed the association between social avoidance and the following adverse outcome profiles: elevated levels of negative self-perception/diminished self-esteem, increased reliance on suppression strategies associated with emotional regulation difficulties, and incidents of peer victimization in educational settings. [79] identified that eighth-grade students'predisposition toward solitude correlates with anxiety, depression, emotional regulation impairments, and reduced self-esteem.

As previously established, in Chinese culture, collectivity and social harmony are considered fundamental values [14]. Consequently, compared to shyness and unsociability, social avoidance may be perceived as the most detrimental form of social withdrawal by children within Chinese cultural contexts [25]. Empirical investigations have demonstrated that social avoidance in early adolescents significantly predicts difficulties in peer interactions and elevated levels of loneliness [24, 27]. Furthermore, with respect to adolescent populations, social avoidance is associated with internalizing problems and peer relationship difficulties, which distinguishes it from other manifestations of social withdrawal [18, 67]. As previously indicated, most of the literature concerning social avoidance pertains exclusively to school-aged children and adolescents [92]. However, investigations focusing on younger children also report substantial adverse outcomes. For instance, [90] identified that social avoidance was positively associated with asocial behavior, peer exclusion, and anxious-fearful behavior among children. Moreover, [91] observed that social avoidance correlates with peer rejection and demonstrates an inverse relationship with preschool prosocial behavior.

Peer relationships are fundamental for appropriate cognitive development and socialization processes [41]. Due to limited opportunities for positive peer interactions, children with low social acceptance typically have restricted chances to acquire normative, adaptive social behaviors and understanding [61]. Regarding comparative analyses, children demonstrated the least interest in initiating social contact with shy and unsociable individuals [13]. Therefore, on one hand, socially avoidant children themselves exhibit reluctance to interact with their peers,on the other hand, other children simultaneously tend to avoid communication with socially avoidant children, which ultimately results in socially avoidant children experiencing significantly reduced peer interaction compared to their counterparts. Consequently, socially avoidant children tend to encounter more substantial challenges in social engagements, particularly within the Chinese cultural context.

### Behavioral Self-Regulation as a Moderator

Researchers have investigated maternal depression symptoms [86], marital conflict [87], household chaos [92], and teacher–child relationships [91] as moderating variables in the relationship between social avoidance and social adjustment. These moderator variables encompass environmental, social interaction, and cognitive factors. To achieve effective self-regulation, a child must utilize cognitive, motivational, and emotional capacities to generate responses that align with contextual expectations [58]. Empirical evidence has demonstrated that effortful and inhibitory control function as protective factors for shy children [69, 89]. According to [58], the diverse abilities associated with behavioral self-regulation are considered components of executive functions but extend beyond these specific processes. It involves the application of all relevant skills within a particular context, suggesting a construct more comprehensive than executive function. Consequently, enhanced behavioral self-regulation may mitigate social adjustment difficulties for socially avoidant children.

Self-regulation involves the deliberate application of one's abilities and characteristics to adapt to dynamic situations, ensuring that verbal and behavioral expressions are appropriately aligned with contextual demands [58]. According to cognitive-behavioral theory [30], individuals encounter various obstacles and challenges when executing behaviors, necessitating regulation and control of their actions. Thus, individuals with developed behavioral self-regulation capabilities are more likely to exhibit appropriate behaviors rather than disrupt social interactions, thereby preventing social adjustment problems. Multiple studies have supported propositions regarding the utility of behavioral self-regulation in relation to children's capacity for academic learning. This evidence indicates that academic adjustment constitutes a principal component of social adjustment as children develop [80]. In a study conducted with a Chinese sample, Georgiou and colleagues established that inhibitory control significantly correlated with reading and mathematical achievement [34]. Research has indicated that behavioral self-regulation exerts significant influence on students'academic achievement in primary education [28]. These researchers further argued that self-regulation demonstrates a positive relationship with social adjustment, particularly in situations involving emotions such as anxiety and embarrassment, as documented by Pecora and colleagues [62]. Therefore, it is essential to investigate how behavioral self-regulation functions to potentially ameliorate difficulties in the social functioning of socially avoidant children.

Children who exhibit social avoidance may reduce their risk of social maladjustment when they possess strong behavioral self-regulation, which functions as a protective factor in their developmental trajectory. Resilience theory focuses on individuals'ability to withstand and adapt to life stressors and adversity [78], with self-regulation serving as a critical internal resource that promotes positive adaptation despite challenging circumstances [54]. According to this framework, behavioral self-regulation functions as both a protective factor and a recovery mechanism that moderates the relationship between social challenges and developmental outcomes. Consequently, the adaptability to social contexts among children with low approach motivation and high avoidance motivation may depend on certain person-specific variables such as behavioral self-regulation. Early childhood represents a developmental period during which most children experience a rapid acquisition process regarding self-control, as substantiated by empirical findings. In this investigation, the head-toes-knees-shoulders (HTKS) tasks were employed to assess children's behavioral self-regulation, which has been conceptualized as a form of"cold"executive function [59]. King et al. [42] determined that peer interactions can facilitate the enhancement of cold cognitive control. Furthermore, research outcomes indicated that children's cold cognition, a manifestation of behavioral regulation, was associated with decreased behavioral concerns in subsequent developmental periods [68]. Conversely, social avoidance constitutes behavior wherein children prefer solitude and actively avoid association with peers [3]. As a result, these children may fail to progress through developmental phases that would facilitate the enhancement of social communication and cognition due to limited peer interaction, thereby increasing vulnerability to social adjustment difficulties. Children who manifest withdrawn behavior frequently experience anxiety and maintain negative self-perceptions [9]. According to self-efficacy theory [5], socially withdrawn children may increasingly avoid social interactions due to persistent negative emotional experiences during interpersonal exchanges, thus exacerbating adjustment problems. In contrast, behavioral self-regulation can attenuate inappropriate verbal and behavioral expressions of socially avoidant children [85], thereby reducing risk factors that contribute to social adjustment challenges. Specifically, elevated behavioral self-regulation enables withdrawn children to more effectively manage their behavior and comprehend others'perspectives. This capability allows them to participate more fully or gradually establish their position within peer interactions as they develop, substantially decreasing the probability of encountering adjustment difficulties. Nevertheless, some scholars present alternative perspectives, asserting that self-regulation can simultaneously exert both positive and negative effects across various child populations. Sawyer et al. [68] observe that enhanced self-regulation may potentially lead to decreased social appropriateness or excessive behavioral control in specific contexts, resulting in withdrawal from social situations. White et al. [83] have identified that elevated levels of inhibitory control can increase the likelihood of anxiety symptom development.

Overall, there has been insufficient attention directed toward the adaptive characteristics of socially avoidant preschoolers between 3 and 6 years of age [92]. However, socially avoidant children may face greater adaptation risks than previously hypothesized [3]. Therefore, examining the relationship between social avoidance and social adjustment remains imperative. Additionally, research has emphasized the importance of attending to children's behavioral self-regulation, particularly during early developmental stages.

The purpose of the current investigation was to examine the relationship among social avoidance, behavioral self-regulation, and social adjustment in young children. We hypothesized that social avoidance would be positively associated with indicators of social adjustment difficulties (asocial behavior, peer exclusion, and anxious-fearful behavior). Furthermore, regarding the moderating effect of behavioral self-regulation, we postulated that positive associations between social avoidance and social adjustment difficulties would be attenuated among children with higher levels of behavioral self-regulation and amplified among those with lower levels of behavioral self-regulation.

## Methods

### Participants

The study sample comprised 221 children aged 42–54 months (111 boys, 52.6%; *M*_*age*_ = 48.81 months, *SD* = 2.51) enrolled in two public kindergartens in Shanghai, China, with class sizes ranging from 25–30 children. For the current investigation, lead teachers from each respective classroom were responsible for assessing children's social adjustment indicators. Maternal reports provided data on children's social avoidance. Data collection occurred through electronic questionnaires, with both maternal and teacher reports providing complete datasets without missing values. Most participants were of Han ethnicity, representing over 90% of the Chinese population and constituting the largest ethnic group in the country. Regarding parental education within these families, approximately 13.5% of fathers and 15.1% of mothers reported having completed only high school education or below. The proportion of parents holding associate degrees was consistent at 21.4% for both fathers and mothers. Bachelor's degrees were held by 32.8% of fathers and 43.2% of mothers. Additionally, 25.5% of fathers and 13.5% of mothers had obtained postgraduate qualifications. To comprehensively address the influence of parental educational attainment, the investigation utilized the mean value of maternal and paternal education levels to represent overall parental education, with higher scores indicating advanced educational attainment [93].

### Procedures

The study protocol received ethical approval from the Ethics Committee of Shanghai Normal University. Following this approval, two public kindergartens in Shanghai were selected as research sites for this investigation. Maternal reports were utilized to assess children's social avoidance behaviors, while teachers completed standardized questionnaires regarding children's social adjustment indicators. The behavioral assessment component involved graduate research assistants demonstrating the head-toes-knees-shoulders task protocol and providing instruction to trained examiners prior to independent administration. Both child participants and examiners conducted the assessment procedures in a controlled, distraction-minimized environment. Data collection procedures were implemented under the supervision of faculty members and graduate students specializing in Chinese early childhood education. Prior to the commencement of formal data collection, informed consent was obtained from both children and their parents through institutional channels, yielding a participation rate of 98%. Data collection was completed during the 2018 academic year.

### Measures

#### Social Avoidance

The mothers provided their responses to the Chinese-adapted CSPS-3, which is commonly used to measure social withdrawal in preschool children [20, 48, 92]. The study concerned a four-item subscale of social avoidance (e.g.,"My child doesn't want to go and mix with other kids. Instead, he or she prefers to be on her own"). Mothers were instructed to provide a rating of 1"absolutely not"to 5"absolutely match". In this study, the researchers used the total item scores, with higher scores indicating more pronounced social avoidance. However, taking into consideration that withdrawn Chinese children also demonstrate similar adjustment patterns, it is worthy to apply statistical adjustments to eliminate the effect of shyness and unsociability while assessing the consequences of social avoidances [67]. Thus, as well as 4 items of the shyness subscale (for example,"My child seems eager to play with others but gets too nervous at times"), The unsociable subscale included 4 items (for instance,"My child likes to play alone and quietly, not with other children"). Previous research has also shown that the CSPS-3 demonstrates good reliability and validity for assessing social avoidance in Chinese preschool children, suggesting it can adequately measure this construct in this population [88, 91]. In this study, the Cronbach’s alpha for shyness, unsociability, and social avoidance were 0.87, 0.67 and 0.72, respectively, which are considered acceptable [81].

#### Behavioral Self-Regulation

The HTKS is commonly used to measure children's behavioral self-regulation skills [63]. In the HTKS, children were told to play a game in which they had to perform the opposite of the response to various oral commands. For example, if the experimenters said,'touch your head', the correct response for the children was to touch their toes. The game was divided into three sections, each with 10 items. In Sect. 1, when the experimenter said'touch your toes', children were required to touch their heads, and vice versa. In Sect. 2, an extra rule was added. Along with the rule from Sect. 1, when the experimenter said'touch your shoulders', children were to touch their knees, and vice versa. In Sect. 3, the rules from previous sections were jumbled. For instance, the correct response to'touch your toes'was touching one’s shoulders. Regarding scoring, the children received two points for a correct response, one for self-correction, and none for incorrect responses. Higher scores mean that they regulate their behavior at a higher level. The results of the earlier studies revealed that the scores of preschool children on the HTKS task were highly correlated with their cognitive flexibility and working memory [56]. In previous studies, the HTKS task has been shown to exhibit good validity and reliability when applied to Chinese preschool children [16, 52, 84]. In this study, the HTKS's Cronbach's alpha was 0.71.

#### Social Adjustment

The educators involved in the study were also able to fill the Chinese version of the CBS [43, 86]. Our study focused mainly on three subscales that include asocial behavior (6 items for instance,"Is a lonely child who has difficulty making friends"), peer exclusion (for instance,"Peer laughter") consisting 7 items, and lastly anxious-fearful behavior (for instance,"Shows signs of sadness, unhappiness, tendency to cry, or dejection"with only 4 items; Cronbach’s α coefficients for the respective subscales was 0.88, 0.89 and 0.75. Previous studies have shown that the CBS has been applied to assess social adjustment among preschoolers and demonstrates reasonable psychometric properties [32, 40, 87].

### Analytic Strategy

Data analysis was conducted using SPSS 22.0 statistical software. Initially, descriptive statistics and bivariate correlation analyses among all primary study variables were calculated, and gender differences in social adjustment indicators (i.e., anxious-fearful behavior, peer exclusion, and asocial behavior) were examined through a series of independent samples t-tests. Subsequently, Hayes's PROCESS macro (Model 1) [39] was employed to investigate the moderating role of behavioral self-regulation in the association between social avoidance and social adjustment outcomes. A moderation effect was determined to be statistically significant if the 95% bias-corrected confidence interval (CI) for the interaction term (social avoidance × behavioral self-regulation) excluded zero [64]. Three independent models were estimated to test the moderating effect of behavioral self-regulation on the associations between social avoidance and (1) anxious-fearful behavior, (2) peer exclusion, and (3) asocial behavior, respectively. To further examine significant interactions, conditional effect analyses were conducted, and interaction effects were graphically represented at high (+ 1 SD) and low (–1 SD) levels of behavioral self-regulation.

## Results

### Preliminary Analyses

Table 1 presents the descriptive statistics and bivariate correlations among study variables. Children's age was negatively and significantly associated with shyness, unsociability, social avoidance, asocial behavior, peer exclusion, and anxious-fearful behavior.

**Table 1:** Descriptive statistics of each variable and the correlation analysis results (*N* = 211)

	1	2	3	4	5	6	7	8	9	10
1. Gender	-									
2. Age	.17^*^	-								
3. Parental education level	.06	.10	-							
4. Shyness	-.08	-.21^**^	.09	-						
5. Unsociability	-.10	-.17^*^	0	.61^**^	-					
6. Social avoidance	-.09	-.34^**^	-.02	.69^**^	.70^**^	-				
7. Asocial behavior	-.16^*^	-.38^**^	.07	.18^*^	.25^**^	.30^**^	-			
8. Peer exclusion	-.22^**^	-.35^**^	-.04	.11	.21^**^	.18^**^	.78^**^	-		
9. Anxious-fearful behavior	-.13	-.48^**^	-.14^*^	.18^*^	.15^*^	.19^**^	.58^**^	.46^**^	-	
10. Behavioral self-regulation	.16^*^	.13^*^	.28^**^	.00	-.08	-.08	-.27^**^	-.32^**^	-.33^**^	-
M	1.47	48.81	2.72	12.56	6.95	5.32	1.18	1.15	1.25	44.79
SD	.50	2.51	1.73	4.82	2.91	2.09	.34	.31	.38	12.89
Skewness	.12	-.52	-.51	.36	.06	.17	2.80	2.86	2.00	−1.42
Kurtosis	-.21	.58	-.51	-.26	.36	1.09	9.22	8.61	3.97	2.44

Gender (boys 1, girls 2); ^*^ *p* <.05; ^**^ *p* <.01; ^***^ *p* <.001

All variables except asocial behavior and peer exclusion exhibited normal distributions, with absolute skewness and kurtosis values for the outcome variables falling below the recommended thresholds of 2 and 7, respectively [82]. Gender differences in anxious-fearful behavior were examined using independent samples t-tests (*M*_*boy*_ = 1.29, *SD* = 0.44; *M*_*girl*_ = 1.20, *SD* = 0.32, *t* = 1.87, *p* < 0.05). Due to the non-normal distributions of asocial behavior and peer exclusion, non-parametric Mann–Whitney U tests were employed, indicating no significant gender differences in asocial behavior (*Z* = −1.92, *p* > 0.05). Conversely, significant gender differences were observed in peer exclusion (*Z* = −2.29, *p* < 0.05).

### The moderating role

**Fig. 1:**
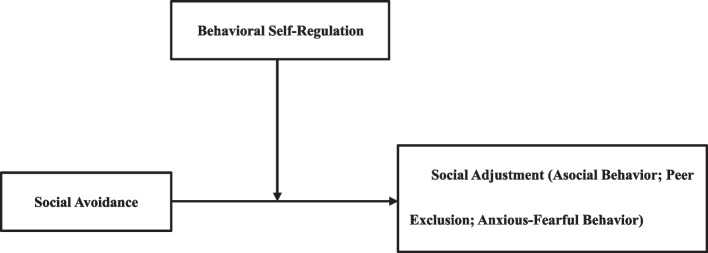
Research model

The subsequent objective was to examine the associations between social avoidance, behavioral self-regulation, and social adjustment outcomes (including asocial behavior, peer exclusion, and anxious-fearful behavior). These analyses controlled for gender, age, parental educational attainment, shyness, and unsociability. To address potential multicollinearity among independent variables, diagnostic indices were examined during regression analyses, confirming the absence of multicollinearity concerns (*Tolerance* > 0.20, *VIF* < 5) [31]. Table 2 summarizes the regression analysis results. Fig. 1

**Table 2:** Effects of social avoidance, behavioral self-regulation in relation to indices of social adjustment

Predictors	*B*	*SE*	*t*	*95%CI*
**Asocial behavior**				
Gender	-.13	.12	−1.13	[-.36,.10]
Age	-.31	.06	−4.94^***^	[-.43, -.18]
Parental education level	.17	.06	2.82^**^	[.05,.29]
Shyness	-.04	.08	-.53	[-.21,.12]
Unsociability	.19	.08	2.30^*^	[.03,.36]
Social avoidance	.01	.10	.14	[-.18,.20]
Behavioral self-regulation	-.20	.06	−3.26^**^	[-.32, -.08]
Social avoidance × Behavioral self-regulation	-.32	.06	−5.41^***^	[-.44, -.20]
* R*^*2*^	.36			
* F*	13.91^***^			
**Peer exclusion**				
Gender	-.23	.13	−1.82	[-.48,.02]
Age	-.30	.06	−4.57^***^	[-.43, -.17]
Parental education level	.07	.06	1.04	[-.06,.19]
Shyness	-.04	.09	-.44	[-.21,.13]
Unsociability	.23	.09	2.58^*^	[.05,.41]
Social avoidance	-.11	.10	−1.08	[-.32,.09]
Behavioral self-regulation	-.25	.07	−3.73^**^	[-.38, -.12]
Social avoidance × Behavioral self-regulation	-.16	.06	−2.53^*^	[-.29, -.04]
* R*^*2*^	.26			
* F*	8.89^***^			
**Anxious-fearful behavior**				
Gender	-.00	.12	-.03	[-.23,.23]
Age	-.45	.06	−7.32^***^	[-.57, -.33]
Parental education level	-.04	.06	-.74	[-.16,.07]
Shyness	.15	.08	1.86	[-.01,.31]
Unsociability	.10	.08	1.14	[-.07,.26]
Social avoidance	-.19	.10	−1.98^*^	[-.38, -.00]
Behavioral self-regulation	-.24	.06	−3.83^***^	[-.36, -.11]
Social avoidance × Behavioral self-regulation	-.22	.06	−3.74^***^	[-.34, -.10]
* R*^*2*^	.36			
* F*	14.23^***^			

^*^ *p* <.05; ^**^ *p* <.01; ^***^ *p* <.001

**Fig. 2:**
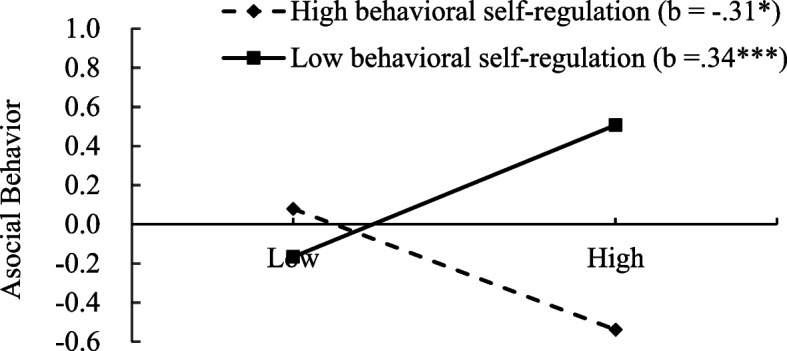
Interaction between behavioral self-regulation and social avoidance on asocial behavior. *p <.05, **p <.01, ***p <.001. The x-axis represents “social avoidance”

Regarding asocial behavior: After controlling for shyness, unsociability, gender, age, and parental educational attainment, social avoidance was not significantly related to asocial behavior (*β* = -.01, *t* =.14, *p* >.05). However, behavioral self-regulation demonstrated a significant negative association with asocial behavior (*β* = -.20, *t* = −3.26, *p* <.01). Additionally, a significant interaction effect between social avoidance and behavioral self-regulation was observed (*β* = -.32, *t* = −5.41, *p* <.001). Conditional effects analysis was conducted to interpret the interaction [2]. As illustrated in Fig. 2, findings indicated that among children with lower behavioral self-regulation, social avoidance was positively associated with asocial behavior (*β* =.34, *SE* =.10, *t* = 3.33, *p* <.001). Conversely, among children with higher behavioral self-regulation, social avoidance was negatively associated with asocial behavior (*β* = −.31, *SE* =.13, *t* = −2.46, *p* <.05).

**Fig. 3:**
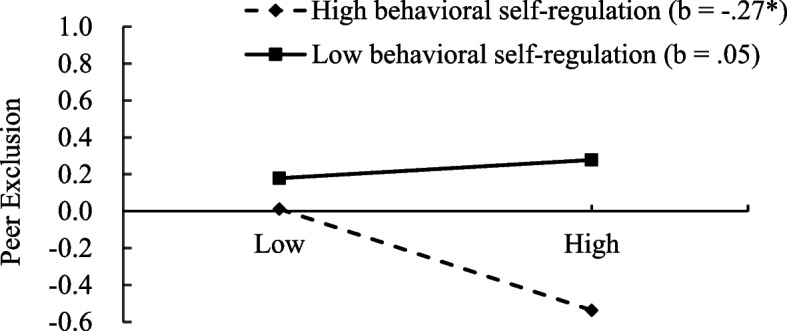
Interaction between behavioral self-regulation and social avoidance on peer exclusion. *p <.05, **p <.01, ***p <.001. The x-axis represents “social avoidance”

Regarding peer exclusion: After adjusting for shyness, unsociability, gender, age, and parental educational attainment, social avoidance was not significantly related to peer exclusion (*β* = -.11, *t* = −1.08, *p* >.05). However, behavioral self-regulation demonstrated a significant negative association with peer exclusion (*β* = -.25, *t* = −3.73, *p* <.01). Furthermore, a significant interaction between social avoidance and behavioral self-regulation was identified (*β* = -.16, *t* = −2.53, *p* <.05). The conditional effects are depicted in Fig. 3. As illustrated, no significant association between social avoidance and peer exclusion was observed among children with lower behavioral self-regulation (*β* =.05, *SE* =.11, *t* =.46, *p* >.05). However, among children with higher behavioral self-regulation, social avoidance was negatively associated with peer exclusion (*β* = -.27, *SE* =.13, *t* = −2.04, *p* <.05).

**Fig. 4:**
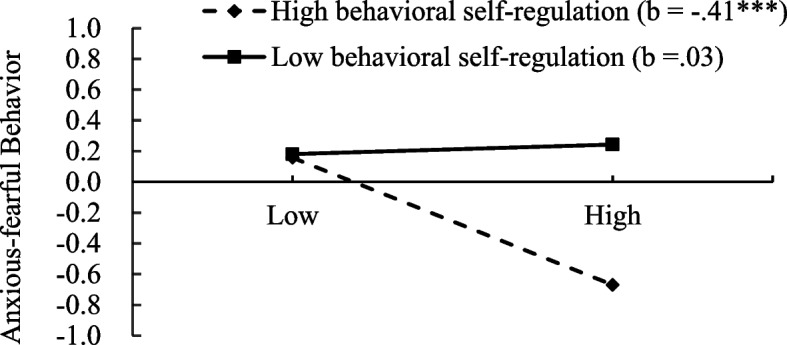
Interaction between behavioral eelf-regulation and social avoidance on anxious-fearful behavior. *p <.05, **p <.01, ***p <.001. The x-axis represents “social avoidance”

Regarding anxious-fearful behavior: After controlling for shyness, unsociability, gender, age, and parental educational attainment, social avoidance was significantly and negatively associated with anxious-fearful behavior (*β* = -.19, *t* = −1.98, *p* <.05). Similarly, behavioral self-regulation demonstrated a significant negative association with anxious-fearful behavior (*β* = -.24, *t* = −3.83, *p* <.001). Additionally, a significant interaction effect between social avoidance and behavioral self-regulation was observed (*β* = -.22, *t* = −3.74, *p* <.001). As illustrated in Fig. 4, no significant association was found between social avoidance and anxious-fearful behavior among children with lower behavioral self-regulation (*β* =.03, *SE* =.10, *t* =.31, *p* >.05). However, among children with higher behavioral self-regulation, social avoidance was negatively associated with anxious-fearful behavior (*β* = −.41, *SE* =.12, *t* = −3.31, *p* <.001).

## Discussion

The present study contributes to the literature by examining the effects of behavioral self-regulation on Chinese kindergarten children’s avoidant behaviors and their adjustment in social contexts. This research established the relationships between social avoidance, behavioral self-regulation, and social adjustment within the framework of the Chinese collectivistic culture.

### Social avoidance and social adjustment

Contrary to our hypothesis, when controlling for shyness, unsociability, gender, age, and parental education level, the predictive effect of social avoidance on asocial behavior is not significant, which diverges from previous findings [90]. This unexpected result may be explained by several factors. First, early childhood represents a developmental stage characterized by egocentrism, wherein peer interactions are neither as frequent nor as profound as those observed during adolescence [77]. Consequently, children displaying social avoidance may not necessarily be perceived as unsociable by their peers. Second, the participants in this study were predominantly middle-class children. Compared with children in the junior class, middle-class children want more attention and praise from their peers, and their behaviors tend to be more peer-oriented [47]. Therefore, social avoidance may not significantly predict asocial or disengaged behaviors in this specific developmental context. Similarly, the predictive effect of social avoidance on peer exclusion is not significant, which contradicts our research hypothesis. The cultural context likely plays a crucial role in this finding. Within Chinese culture, where collective harmony is emphasized [14], adults may actively intervene to prevent explicit peer exclusion. This cultural moderation could attenuate the natural relationship between social avoidance and peer exclusion that might otherwise emerge. From a methodological perspective, while mothers provide valuable insights about their children's social avoidance tendencies, they may have limited visibility into the actual peer dynamics within the classroom setting. Social avoidance behaviors observed by mothers at home may represent an incomplete picture that doesn't accurately reflect children's social interactions in educational environments. Future research should consider employing multiple assessment methods and informants to evaluate social avoidance and peer exclusion comprehensively across different settings. Additionally, this finding suggests that the relationship between social avoidance and peer exclusion likely involves complex mediational or moderating pathways rather than a direct effect. For instance, factors such as household chaos [92], teacher–child relationships [91], and children's effortful control [69], may influence whether socially avoidant children experience peer exclusion behaviors. Our significant interaction findings with behavioral self-regulation support this complexity, indicating that individual characteristics influence how social avoidance relates to social adjustment outcomes.

Our findings reveal complex relationships between social avoidance and various indicators of social adjustment. While social avoidance has been identified as having the most deleterious impact on social adjustment compared to other forms of social withdrawal [21, 90], these relationships may be affected by multiple interacting factors rather than simple linear relationships [87, 91]). Therefore, identifying beneficial moderating variables in the relationship between social avoidance and social adjustment is particularly important. This study focuses on behavioral self-regulation as a potential protective moderator in the relationship between social avoidance and social adjustment outcomes.

### The moderating role of behavioral self-regulation

Effective behavioral self-regulation is essential for accomplishing adaptive developmental tasks throughout all stages of life [54]. Our analysis confirms our hypotheses, revealing that behavioral self-regulation moderates the relations between social avoidance and social adjustment, including asocial behavior, anxious-fearful behavior, and peer exclusion. These findings align with existing research demonstrating the protective function of behavioral self-regulation among socially avoidant kindergarten children [69, 89], which suggested that behavioral self-regulation may partially mitigate the risk of social maladjustment in children who exhibit social avoidance.

We first examine peer exclusion outcomes. Our analysis revealed a significant interaction effect between social avoidance and behavioral self-regulation, demonstrating that behavioral self-regulation serves as a significant moderator in this relationship. This finding is consistent with previous research. For instance, higher levels of behavioral self-regulation are associated with enhanced social competence [58], fewer peer relationship problems [65], and greater self-realization [57]. The moderating effect can be effectively interpreted through the lens of resilience theory, which emphasizes that individuals possess internal resources to adapt positively despite challenging circumstances [78]. Within this theoretical framework, behavioral self-regulation appears to function as a key resilience factor that helps children navigate social challenges more effectively. Thus, socially avoidant children with stronger behavioral self-regulation abilities are better able to foster peer acceptance and maintain their physical and mental well-being. Conversely, poor behavioral self-regulation may prevent children from making appropriate behavioral choices, leading to social exclusion [73, 76], aggression [38], and antisocial behavior [76]. Hence, socially avoidant children with inadequate behavioral self-regulation abilities face significant adjustment challenges. They not only have limited peer interactions but also struggle to effectively mobilize their emotions, cognition, and other resources to respond appropriately in social situations. This implies that a high level of behavioral regulation ability during social interaction reduces peer rejection among socially avoidant children.

Next, we examine how behavioral self-regulation moderates the relationship between social avoidance and anxious-fearful behavior. Among children with lower levels of behavioral self-regulation, social avoidance showed a positive but non-significant association with anxious-fearful behavior. In contrast, among children with higher levels of behavioral self-regulation, social avoidance showed a significant negative association with anxious-fearful behavior.

To understand these findings, it is important to consider the broader context of internalizing problems in children. Internalizing problems encompass various emotional and behavioral difficulties directed inward, including both anxious-fearful behaviors and depressive symptoms [70]. These internalizing problems are often characterized by maladaptive information processing and cognitive patterns [22]. Children experiencing internalizing difficulties frequently demonstrate heightened attention to threatening stimuli and difficulty disengaging their attention once it is captured [17]. Behavioral self-regulation assists children in maintaining focus, retaining instructions, and completing tasks despite environmental distractions [11]. Importantly, behavioral self-regulation is associated with executive functions, which research indicates may serve as predisposing risk factors, maintenance elements, and/or adverse outcomes of various internalizing problems [71]. The moderating effect of behavioral self-regulation may be explained by its influence on cognitive processes underlying internalizing problems. Children with stronger behavioral self-regulation abilities can better manage their attention and emotional responses, potentially reducing their vulnerability to anxious-fearful behaviors and other internalizing symptoms [37]. Additionally, research has shown that negative cognitive patterns play an important mediating role in the development of internalizing problems in children [1]. Supporting our findings, research on adolescents revealed that those with robust behavioral self-regulation skills exhibited significantly better adaptive functioning across multiple domains, such as social competence, academic performance, problem behaviors, and various internalizing symptoms [8].

Finally, we examined behavioral self-regulation as a moderator of the relationship between social avoidance and asocial behavior. Among children with less developed behavioral self-regulation abilities, we found a substantial positive association between social avoidance and asocial behavior. In contrast, children with more advanced behavioral self-regulation exhibited a significant negative relationship between social avoidance and asocial behavior. Previous research has identified emotion regulation as a moderator of the relationship between social avoidance and asocial behavior. For instance, in children with lower emotion regulation, the association between social avoidance and asociality was negative, whereas this pattern was not observed in children with higher levels of emotion regulation [88]. Hence, considering the significant role of behavioral self-regulation in regulating emotions, it is plausible that behavioral self-regulation also serves as a further moderator of asocial behavior in social avoidance children. The frontal brain regions play a pivotal role in behaviors crucial to behavioral self-regulation [23]. Blumer and Benson [6] identified two categories of personality changes commonly observed following prefrontal lobe injury: pseudodepressive personality and pseudopsychotic personality. Pseudodepressive personality, characterized by apathy, diminished motivation, cognitive impairment, and impaired planning ability, is linked to injury in the dorsolateral region of the frontal lobe [33, 74]. Furthermore, based on the social skills model [12], socially avoidant children may lack the motivation to engage in social interactions, potentially leading to missed opportunities for learning social skills from peers and subsequent social adjustment difficulties. Nonetheless, children with strong behavioral self-regulation abilities can exert better behavioral control and obtain more social support, which may explain the negative association between social avoidance and asocial behavior in this group.

Among children with low levels of behavioral self-regulation, a significant positive association exists between social avoidance and asocial behavior. This distinction may be explained by the classification of behavioral self-regulation as a"cold"cognitive ability [59] that has been shown to predict early literacy competencies [53], as well as vocabulary development and mathematical performance in children [55]. In contrast,"hot"self-regulation, such as emotional regulation, more directly modulates the balance between social rejection and social integration [88]. While"cold"cognitive abilities primarily influence academic performance, they may also indirectly affect children's social adjustment [53]. A longitudinal study shows that asocial beliefs significantly and positively predict asocial behavior [36]. When children with low behavioral self-regulation encounter social difficulties, these asocial beliefs may more readily translate into avoidant behaviors, further reinforcing their separation from peers and intensifying social avoidance. Research suggests peer rejection may develop through intermediate processes rather than being directly caused by social avoidance. In one longitudinal study, researchers observed that social exclusion leads to children's poor self-regulation, whereas lack of self-regulation, in turn, enhances the risk of exclusion [73].

### Limitations and suggestions for further research

Our findings suggest that socially avoidant children in Chinese kindergarten who have strong behavioral self-regulation can use this resource as a protective mechanism to enhance their social acceptance. However, several limitations of the present study should be acknowledged. First, data collection was limited to two public kindergartens in Shanghai, China, potentially restricting the generalizability of our findings to other regions. The study's findings should be interpreted with caution, since Shanghai is a relatively developed region in China. Future studies would benefit from a broader geographical coverage, including urban and rural areas across China. Second, the assessment of social avoidance may be influenced by cultural values; cross-cultural studies are necessary to validate whether these mechanisms are applicable across different cultural contexts. Third, due to the cross-sectional nature of our study design, the causality and directionality of the relationships between variables cannot be conclusively established. Bidirectional associations might exist—for instance, social adjustment difficulties could influence social avoidance rather than simply the reverse. Therefore, caution should be exercised when interpreting these findings, and further longitudinal research is needed to clarify the temporal sequences and establish causal relationships among social avoidance, behavioral self-regulation, and social adjustment. Finally, this study focused on behavioral self-regulation as a moderator in the relationship between social avoidance and social adjustment outcomes. Future research should incorporate additional variables to enhance our understanding of this phenomenon.

## Conclusions

After controlling for relevant variables (child gender, age, parental education level, shyness, and unsociability), this study suggests that behavioral self-regulation may moderate the relationship between social avoidance and social adjustment (asocial behavior, peer exclusion, and anxious-fearful behavior). Specifically, for children with higher levels of behavioral self-regulation, social avoidance was negatively associated with asocial behavior, peer exclusion, and anxious-fearful behaviors. For children with lower behavioral self-regulation, social avoidance was positively associated with asocial behavior while showing no significant associations with peer exclusion and anxious-fearful behaviors. These findings highlight the protective function of behavioral self-regulation in mitigating the negative association between social avoidance and social adjustment. They emphasize the importance for educators and parents to develop children's self-regulatory abilities while providing targeted support for asocial behaviors.

## Data Availability

The identified datasets analyzed during the current study are available from the corresponding author on reasonable request.

## Abbreviations

CSPS-3: The Third Version of Child Social Preference Scale
CBS: Child Behavior Scale
HTKS: Head-Toes-Knees-Shoulders tasks
